# Supplementary material for: Extensive genomic study characterizing three Paracoccaceae populations and revealing Pseudogemmobacter lacusdianii sp. nov. and Paracoccus broussonetiae sp. nov
Source: Microbiol Spectr. 2024 Sep 27;12(11):e01088-24. doi: 10.1128/spectrum.01088-24 (PMC11537045; doi:10.1128/spectrum.01088-24)
Supplement: Supplemental material — Tables S1 to S4; Fig. S1 to S6. [file spectrum.01088-24-s0001.docx]

**Extensive genomic study characterizing three *Paracoccaceae* populations and revealing *Pseudogemmobacter lacusdianii* sp. nov. and *Paracoccus broussonetiae* sp. nov.**

# Yang Deng^1†^, Cong-Jian Li^1†^, Jing Zhang^2^, Wei-Hong Liu^3^, Li-Yan Yu^1^, Yu-Qin Zhang^1*^

^1^Institute of Medicinal Biotechnology, Chinese Academy of Medical Sciences & Peking Union Medical College, Beijing 100050, P. R. China

^2^[Southern Marine Science and Engineering Guangdong Laboratory, Guangzhou, 511458, P. R. China](https://www.microbiologyresearch.org/search?option1=pub_affiliation&value1=)

^3^Yunnan Provincial Key Laboratory of Entomological Biopharmaceutical R&D, Dali University, Dali 671003, P. R. China.

^†^These authors have contributed equally to this work and share first authorship

**^*^**Author for correspondence:

Yu-Qin Zhang

Tel: +86-10-83167110

Fax: +86-10-83167110

1. Mail: yzhang@imb.pumc.edu.cn

**Figure S1.** Transmission electron microscopy images from cells of strains (a) CPCC 101601^T^, (b) CPCC 101403^T^ and (c) CPCC 100767


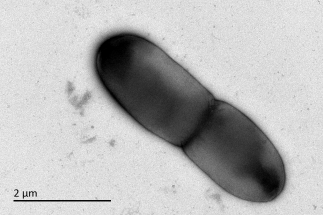

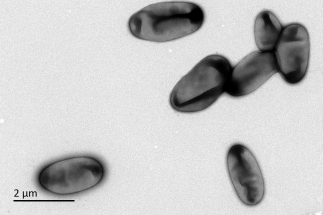

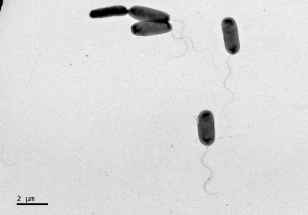


(c)

(b)

(a)

**Figure S2.** 5-ALA standard curve and the absorbance values of the strain CPCC 101601^T^ in its respective fermentation broth at 553 nm.


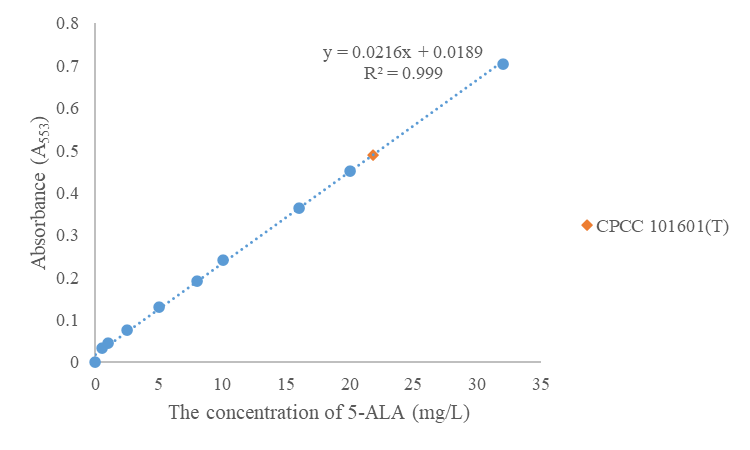


**Figure S3.** Polar lipid profiles for strains CPCC 101601^T^, CPCC 101403^T^ and CPCC 100767 after separation by two-dimensional TLC.

Detection is shown after spraying with (a) molybdatophosphoric acid reagent; (b) ninhydrin stain reagent (c); molybdenum blue stain reagent; and (d) p-anisaldehyde stain reagent.

DPG, diphosphatidylglycerol; PE, phosphatidylethanolamine; PME, phosphatidylmonomethylethanolamine; PG, phatidylglycerol; PC, phosphatidylcholine; AL,unidentified aminolipids; GL1-GL3, unidentified glycolipids; L, unidentified lipid.


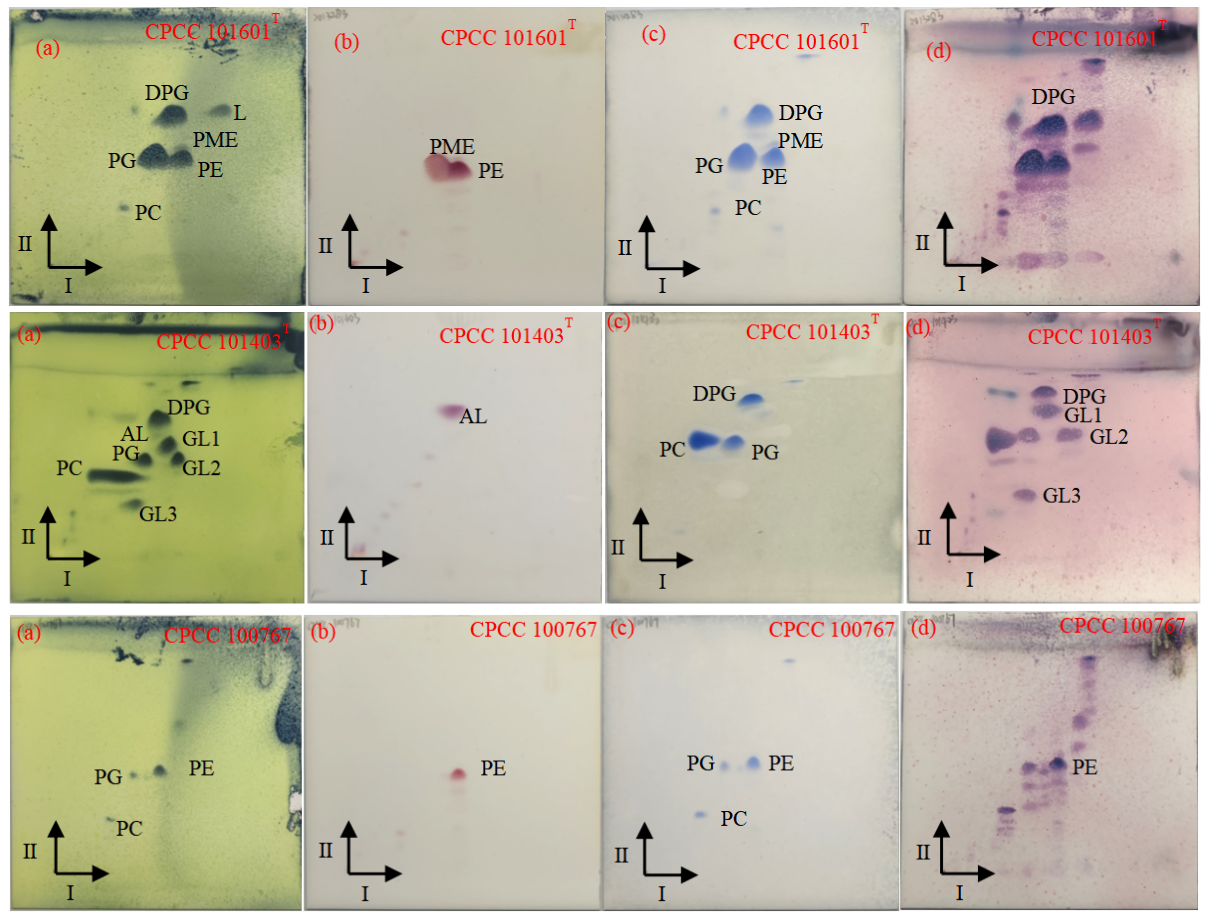


**Figure S4.** Maximum-Likelihood phylogenomic tree showing the phylogenetic positions of strains CPCC 101601^T^, CPCC 101403^T^ and CPCC 100767 within the family *Paracoccaceae* based on conserved core genes.

Bootstrap values are shown at branch nodes as a percentage of 1,000 bootstrap replicates. RefSeq assembly accession numbers for *Paracoccaceae* references are shown in brackets. *Roseobacter denitrificans* ATCC 33942^T^ (RefSeq assembly accession no. GCF_900113215.1) was used as the outgroup. Bar indicates 0.05 substitutions per nucleotide position.


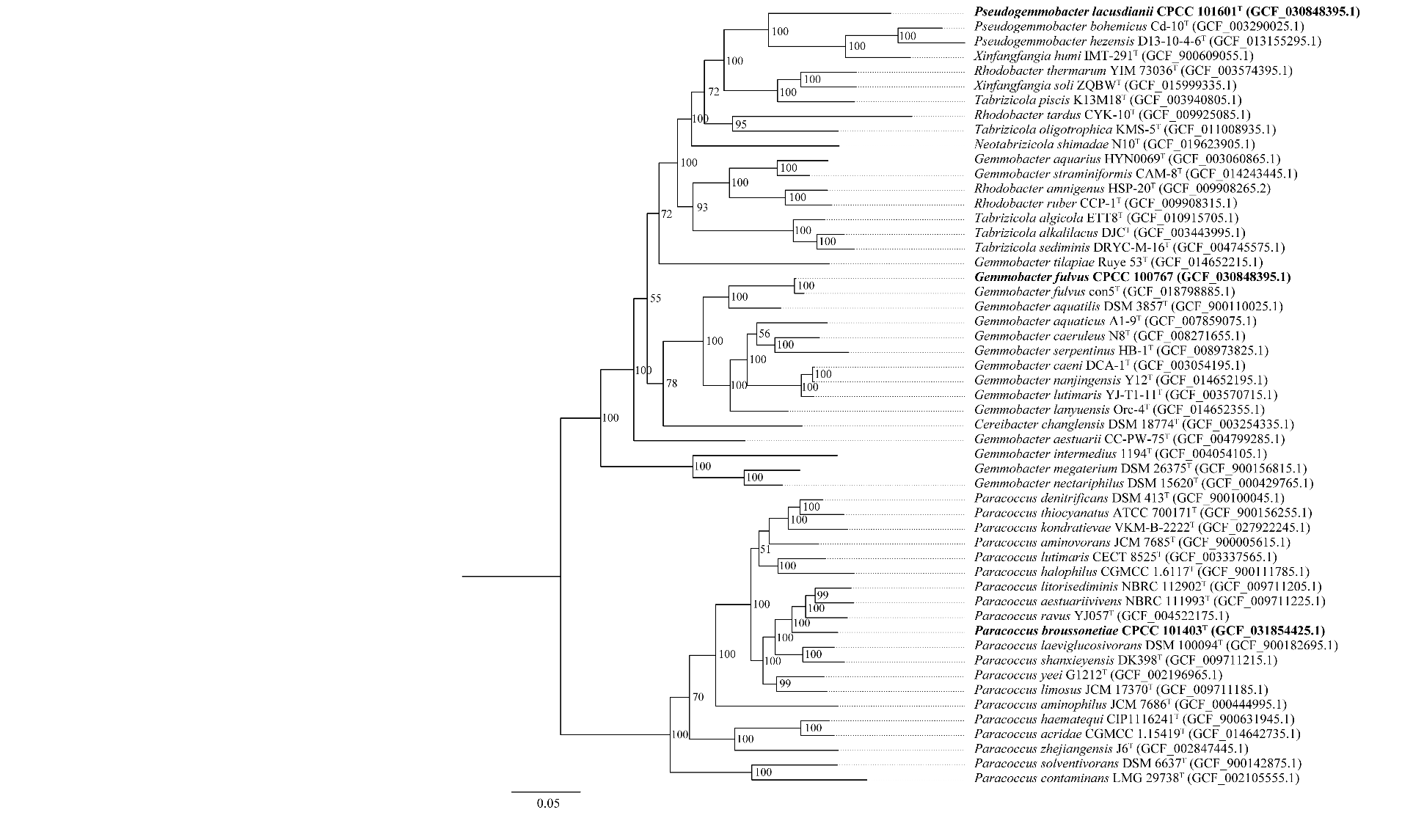


**Table S1**. The 16S rRNA gene sequence similarities (%) between strains CPCC 101601^T^, CPCC 101403^T^, CPCC 100767 and other closely related strains of the family *Paracoccaceae*.

Strains: 1, CPCC 101601^T^; 2, CPCC 101403^T^; 3, CPCC 100767; 4, *Pseudogemmobacter bohemicus* Cd-10^T^; 5, *Pseudogemmobacter hezensis* D13-10-4-6^T^; 6, *Xinfangfangia humi* IMT-291^T^; 7, *Xinfangfangia soli* ZQBW^T^; 8, *Gemmobacter fulvus* JCM 34791^T^; 9, *Gemmobacter aquatilis* DSM 3857^T^; 10, *Gemmobacter fontiphilus* LMG 25376^T^; 11, *Gemmobacter lutimaris* JCM 32828^T^; 12, *Gemmobacter tilapiae* KCTC 23310^T^; 13, *Gemmobacter aquarius* NBRC 113115^T^; 14, *Gemmobacter serpentinus* NBRC 113115^T^; 15, *Gemmobacter caeni* DSM 21823^T^; 16, *Gemmobacter aquaticus* NBRC 104254^T^; 17, *Gemmobacter lanyuensis* KCTC 23714^T^; 18, *Gemmobacter straminiformis* JCM 31905^T^; 19, *Gemmobacter caeruleus* N8^T^; 20, *Gemmobacter nanjingensis* KCTC 23298^T^; 21, *Gemmobacter aestuarii* JCM 19754^T^; 22, *Gemmobacter intermedius* 119/4^T^; 23, *Gemmobacter megaterium* JCM 18498^T^, 24, *Gemmobacter nectariphilus* NBRC 100046^T^; 25, *Paracoccus yeei* ATCC BAA-599^T^; 26, *Paracoccus lutimaris* HDM-25^T^; 27, *Paracoccus aestuariivivens* GHD-30^T^. 16S rRNA gene nucleotide sequence similarities (%) > 98.65% are highlighted in bold.

|  | 1 | 2 | 3 | 4 | 5 | 6 | 7 | 8 | 9 | 10 | 11 | 12 | 13 | 14 | 15 | 16 | 17 | 18 | 19 | 20 | 21 | 22 | 23 | 24 | 25 | 26 | 27 |
| --- | --- | --- | --- | --- | --- | --- | --- | --- | --- | --- | --- | --- | --- | --- | --- | --- | --- | --- | --- | --- | --- | --- | --- | --- | --- | --- | --- |
| 1 | **100** |  |  |  |  |  |  |  |  |  |  |  |  |  |  |  |  |  |  |  |  |  |  |  |  |  |  |
| 2 | 92.9 | **100** |  |  |  |  |  |  |  |  |  |  |  |  |  |  |  |  |  |  |  |  |  |  |  |  |  |
| 3 | 94.7 | 94.6 | **100** |  |  |  |  |  |  |  |  |  |  |  |  |  |  |  |  |  |  |  |  |  |  |  |  |
| 4 | 96.0 | 92.7 | 93.9 | **100** |  |  |  |  |  |  |  |  |  |  |  |  |  |  |  |  |  |  |  |  |  |  |  |
| 5 | 96.8 | 93.2 | 94.0 | 97.7 | **100** |  |  |  |  |  |  |  |  |  |  |  |  |  |  |  |  |  |  |  |  |  |  |
| 6 | 96.4 | 92.9 | 94.1 | **99.2** | 97.5 | **100** |  |  |  |  |  |  |  |  |  |  |  |  |  |  |  |  |  |  |  |  |  |
| 7 | 95.4 | 93.6 | 94.1 | 96.6 | 95.4 | 97.3 | **100** |  |  |  |  |  |  |  |  |  |  |  |  |  |  |  |  |  |  |  |  |
| 8 | 94.4 | 94.4 | **99.9** | 93.8 | 94.0 | 94.0 | 94.1 | **100** |  |  |  |  |  |  |  |  |  |  |  |  |  |  |  |  |  |  |  |
| 9 | 94.7 | 94.2 | 98.1 | 93.6 | 93.9 | 93.8 | 94.3 | 98.0 | **100** |  |  |  |  |  |  |  |  |  |  |  |  |  |  |  |  |  |  |
| 10 | 94.7 | 93.3 | 97.4 | 93.6 | 94.0 | 93.7 | 93.7 | 97.1 | 98.0 | **100** |  |  |  |  |  |  |  |  |  |  |  |  |  |  |  |  |  |
| 11 | 94.0 | 94.2 | 97.3 | 93.6 | 93.9 | 93.6 | 94.2 | 97.4 | 98.6 | 98.5 | **100** |  |  |  |  |  |  |  |  |  |  |  |  |  |  |  |  |
| 12 | 94.5 | 94.3 | 97.3 | 93.6 | 93.8 | 93.8 | 94.6 | 97.3 | 97.6 | 96.8 | 97.2 | **100** |  |  |  |  |  |  |  |  |  |  |  |  |  |  |  |
| 13 | 93.6 | 94.6 | 96.5 | 93.8 | 93.7 | 94.0 | 94.6 | 96.5 | 97.1 | 96.2 | 96.5 | 96.5 | **100** |  |  |  |  |  |  |  |  |  |  |  |  |  |  |
| 14 | 94.1 | 93.7 | 96.5 | 93.8 | 93.8 | 94.0 | 94.2 | 97.0 | 97.5 | 97.6 | 98.0 | 95.8 | 96.0 | **100** |  |  |  |  |  |  |  |  |  |  |  |  |  |
| 15 | 93.4 | 94.7 | 96.3 | 93.0 | 94.3 | 93.5 | 94.5 | 96.3 | 97.0 | 97.2 | 98.2 | 96.3 | 96.3 | 96.6 | **100** |  |  |  |  |  |  |  |  |  |  |  |  |
| 16 | 93.5 | 93.3 | 96.3 | 92.8 | 93.4 | 93.1 | 93.6 | 97.1 | 97.5 | 98.0 | 97.9 | 95.6 | 95.8 | 98.0 | 96.3 | **100** |  |  |  |  |  |  |  |  |  |  |  |
| 17 | 93.1 | 93.5 | 96.2 | 93.2 | 94.0 | 93.6 | 94.0 | 96.3 | 97.4 | 97.6 | 97.9 | 96.4 | 96.5 | 97.0 | 97.0 | 97.5 | **100** |  |  |  |  |  |  |  |  |  |  |
| 18 | 93.5 | 94.3 | 96.0 | 93.8 | 93.7 | 94.2 | 94.5 | 96.1 | 96.5 | 95.5 | 96.0 | 96.3 | 98.4 | 95.3 | 95.3 | 94.9 | 95.8 | **100** |  |  |  |  |  |  |  |  |  |
| 19 | 93.4 | 93.8 | 95.9 | 93.1 | 93.9 | 93.5 | 94.2 | 96.6 | 97.1 | 96.6 | 97.0 | 95.2 | 95.5 | 96.9 | 95.4 | 98.0 | 97.1 | 95.1 | **100** |  |  |  |  |  |  |  |  |
| 20 | 93.8 | 94.4 | 95.8 | 93.2 | 94.3 | 93.8 | 94.8 | 96.4 | 97.2 | 97.3 | 98.4 | 95.6 | 95.6 | 95.8 | **99.1** | 95.6 | 96.4 | 95.5 | 95.7 | **100** |  |  |  |  |  |  |  |
| 21 | 93.7 | 94.0 | 94.8 | 93.9 | 94.0 | 94.5 | 95.3 | 95.1 | 95.8 | 94.9 | 95.3 | 95.4 | 95.1 | 95.3 | 95.4 | 94.5 | 95.7 | 95.8 | 94.9 | 94.7 | **100** |  |  |  |  |  |  |
| 22 | 95.3 | 92.5 | 94.5 | 95.3 | 95.8 | 95.4 | 94.5 | 94.4 | 94.1 | 93.5 | 94.2 | 95.0 | 93.3 | 93.4 | 94.0 | 93.5 | 93.6 | 93.5 | 93.6 | 94.5 | 93.6 | **100** |  |  |  |  |  |
| 23 | 94.1 | 95.0 | 95.8 | 93.5 | 94.2 | 93.7 | 94.8 | 95.7 | 95.2 | 94.5 | 95.2 | 96.4 | 95.7 | 95.3 | 94.9 | 94.7 | 94.7 | 95.3 | 95.7 | 94.6 | 95.9 | 94.3 | **100** |  |  |  |  |
| 24 | 94.1 | 94.0 | 95.4 | 95.3 | 93.8 | 95.4 | 95.8 | 95.3 | 95.3 | 94.6 | 94.9 | 96.0 | 95.9 | 94.8 | 94.3 | 94.3 | 94.8 | 96.0 | 95.1 | 94.5 | 95.7 | 94.7 | 96.5 | **100** |  |  |  |
| 25 | 92.5 | 98.3 | 94.2 | 92.9 | 92.8 | 93.3 | 94.6 | 94.2 | 94.4 | 93.5 | 94.5 | 94.6 | 94.7 | 93.7 | 94.6 | 93.5 | 94.2 | 94.3 | 94.4 | 94.4 | 94.9 | 92.8 | 94.9 | 94.5 | **100** |  |  |
| 26 | 92.5 | 98.3 | 93.5 | 92.5 | 93.2 | 92.9 | 93.6 | 93.5 | 93.4 | 92.8 | 93.6 | 94.5 | 94.7 | 93.2 | 94.2 | 92.7 | 93.2 | 94.2 | 93.0 | 94.3 | 93.8 | 92.8 | 94.8 | 94.2 | 97.3 | **100** |  |
| 27 | 93.5 | 97.7 | 93.5 | 93.7 | 94.0 | 94.0 | 94.5 | 94.0 | 93.3 | 92.8 | 93.7 | 94.3 | 94.4 | 93.3 | 93.5 | 93.0 | 93.0 | 94.2 | 93.0 | 93.8 | 92.8 | 93.9 | 94.2 | 94.1 | 96.6 | 97.6 | **100** |

**Table S2**. Genome features of strains CPCC 101601^T^, CPCC 101403^T^, CPCC 100767, and their closely related strains.

Strains: 1, CPCC 101601^T^; 2, *Pseudogemmobacter bohemicus* Cd-10^T^; 3, *Pseudogemmobacter hezensis* D13-10-4-6^T^; 4, *Xinfangfangia humi* IMT-291^T^; 5, CPCC 100767; 6, *Gemmobacter fulvus* con5^T^; 7, CPCC 101403^T^; 8, *Paracoccus yeei* ATCC BAA-599^T^.

| **Characteristic** | **1** | **2** | **3** | **4** | **5** | **6** | **7** | **8** |
| --- | --- | --- | --- | --- | --- | --- | --- | --- |
| No. of contigs | 50 | 88 | 66 | 147 | 32 | 7 | 68 | 199 |
| Genome size  (Mbp) | 3.8 | 5.6 | 4.6 | 4.5 | 4.3 | 4.7 | 4.8 | 4.4 |
| G+C (%) | 61.0 | 63.0 | 62.5 | 66.5 | 64.0 | 64.0 | 64.0 | 67.0 |
| N50 Length (bp) | 213,745 | 133,464 | 201,239 | 71,352 | 213,745 | - | 236,368 | 57,248 |
| No. of CDS | 3,544 | 5,219 | 4,325 | 4,327 | 4,149 | 4,460 | 4,620 | 4,249 |
| DDBJ/EMBL/Ge  nbnk accession  number of draft genome | JAVDBT000000000 | QNHG00000000 | JABJXT010000000 | UXAW00000000 | JAVCWG000000000 | CP076361 | JAVRQI000000000 | NIPZ00000000 |
| GenBank/RefSeq assembly accession number | GCF_030848395.1 | GCF_003290025.1 | GCF_013155295.1 | GCF_900609055.1 | GCF_030848395.1 | GCF_018798885.1 | GCF_031854425.1 | GCF_002196965.1 |

CDSs, coding sequences.

**Table S3**. Average nucleotide identity (ANI) and digital DNA–DNA hybridization (dDDH)values (%) between strains CPCC 101601^T^, CPCC 101403^T^, CPCC 100767, and other closely related strains of the family *Paracoccaceae*.

Strains: 1, CPCC 101601^T^; 2, CPCC 101403^T^; 3, CPCC 100767; 4, *Pseudogemmobacter bohemicus* Cd-10^T^; 5, *Pseudogemmobacter hezensis* D13-10-4-6^T^; 6, *Xinfangfangia humi* IMT-291^T^; 7, *Xinfangfangia soli* ZQBW^T^; 8, *Gemmobacter fulvus* JCM 34791^T^; 9, *Gemmobacter aquatilis* DSM 3857^T^; 10, *Gemmobacter lutimaris* JCM 32828^T^; 11, *Gemmobacter tilapiae* KCTC 23310^T^; 12, *Gemmobacter aquarius* NBRC 113115^T^; 13, *Gemmobacter serpentinus* NBRC 113115^T^; 14, *Gemmobacter caeni* DSM 21823^T^; 15, *Gemmobacter aquaticus* NBRC 104254^T^; 16, *Gemmobacter lanyuensis* KCTC 23714^T^; 17, *Gemmobacter straminiformis* JCM 31905^T^; 18, *Gemmobacter caeruleus* N8^T^; 19, *Gemmobacter nanjingensis* KCTC 23298^T^; 20, *Gemmobacter aestuarii* JCM 19754^T^; 21, *Gemmobacter intermedius* 119/4^T^; 22, *Gemmobacter megaterium* JCM 18498^T^, 23, *Gemmobacter nectariphilus* NBRC 100046^T^; 24, *Paracoccus yeei* ATCC BAA-599^T^; 25, *Paracoccus lutimaris* HDM-25^T^; 26, *Paracoccus aestuariivivens* GHD-30^T^. ANI values > 95% and dDDH values > 70% are highlighted in bold.

| Strain | ANI value (%) |  |  | dDDH value (%) |  |  |
| --- | --- | --- | --- | --- | --- | --- |
|  | 1 | 2 | 3 | 1 | 2 | 3 |
| 1 | **100** |  |  | **100** |  |  |
| 2 | 71.3 | **100** |  | 18.8 | **100** |  |
| 3 | 73.4 | 72.5 | **100** | 18.3 | 18.6 | **100** |
| 4 | 74.6 | 71.9 | 73.6 | 21.0 | 18.7 | 19.5 |
| 5 | 74.5 | 72.2 | 73.8 | 20.3 | 19.6 | 19.4 |
| 6 | 74.7 | 72.6 | 74.6 | 20.1 | 19.0 | 18.9 |
| 7 | 74.3 | 72.8 | 74.8 | 19.0 | 19.2 | 16.6 |
| 8 | 73.0 | 72.5 | **98.5** | 18.5 | 18.9 | **87.4** |
| 9 | 73.4 | 72.7 | 80.6 | 18.9 | 19.2 | 22.9 |
| 10 | 73.4 | 72.6 | 78.3 | 19.0 | 19.8 | 21.1 |
| 11 | 71.9 | 71.8 | 73.7 | 18.5 | 19.1 | 19.0 |
| 12 | 72.9 | 71.2 | 75.3 | 18.9 | 19.2 | 19.8 |
| 13 | 73.5 | 72.5 | 77.5 | 19.3 | 19.3 | 20.6 |
| 14 | 73.1 | 72.6 | 78.3 | 18.8 | 19.6 | 20.9 |
| 15 | 72.5 | 72.4 | 76.7 | 18.5 | 19.4 | 20.0 |
| 16 | 72.9 | 72.8 | 78.2 | 18.5 | 19.4 | 20.5 |
| 17 | 73.3 | 73.2 | 75.3 | 18.6 | 19.3 | 19.8 |
| 18 | 73.4 | 72.4 | 78.7 | 18.9 | 19.2 | 21.1 |
| 19 | 73.2 | 72.8 | 78.1 | 18.6 | 19.8 | 21.0 |
| 20 | 73.2 | 73.3 | 76.1 | 19.2 | 19.3 | 19.5 |
| 21 | 72.7 | 71.5 | 72.8 | 20.9 | 19.5 | 18.9 |
| 22 | 72.2 | 72.3 | 74.3 | 19.4 | 19.7 | 19.3 |
| 23 | 72.9 | 73.2 | 75.1 | 19.3 | 19.4 | 19.4 |
| 24 | 71.6 | 80.3 | 73.4 | 19.2 | 22.8 | 19.3 |
| 25 | 71.7 | 79.2 | 73.4 | 18.8 | 25.2 | 19.0 |
| 26 | 71.3 | 79.6 | 72.1 | 18.7 | 22.3 | 14.1 |

**Table S4**. Abundance of genes implicated in taxonomic characterization found in the genomes of strain CPCC 101601^T^, CPCC 101403^T^, and CPCC 100767.

Strains: 1, CPCC 101601^T^; 2, CPCC 101403^T^; 3, CPCC 100767.

| **Gene symbol** | **Product** | **Copy number of** | | |
| --- | --- | --- | --- | --- |
|  |  | **CPCC 101601^T^** | **CPCC 101403^T^** | **CPCC 100767** |
| **Lipid metabolism** |  |  |  |  |
| *AccB* | biotin carboxyl carrier protein /biotin carboxylase [6.4.1.2] | 1 | 1 | 2 |
| *AccC* | acetyl-CoA carboxylase, biotin carboxylase subunit [6.4.1.2] | 1 | 1 | 3 |
| *AccD* | acetyl-CoA carboxylase carboxyl transferase subunit beta [6.4.1.2] | 1 | 1 | 1 |
| *fabA* | 3-hydroxyacyl-[acyl-carrier protein] dehydratase/trans-2-decenoyl-[acyl-carrier protein] isomerase | 1 | 1 | 1 |
| *fabB* | 3-oxoacyl-[acyl-carrier-protein] synthase I | 2 | 1 | 1 |
| *fabD* | [acyl-carrier-protein] S-malonyltransferase | 2 | 1 | 2 |
| *fabG* | 3-oxoacyl-[acyl-carrier protein] reductase | 6 | 7 | 8 |
| *fabH* | 3-oxoacyl-[acyl-carrier-protein] synthase III | 2 | 2 | 5 |
| *fabI* | enoyl-[acyl-carrier protein] reductase I | 2 | 2 | 2 |
| *fabK* | enoyl-[acyl-carrier protein] reductase II | 0 | 0 | 1 |
| *fabZ* | 3-hydroxyacyl-[acyl-carrier-protein] dehydratase | 1 | 1 | 1 |
| **Glycerophospholipid metabolism** |  |  |  |  |
| *pssA* | phosphatidylserine synthase | 1 | 0 | 1 |
| *pgpA* | phosphatidylglycerophosphatase A | 1 | 1 | 1 |
| *pgsA* | phosphatidylglycerol synthase | 1 | 1 | 1 |
| *pcs* | phosphatidylcholine synthase | 1 | 1 | 1 |
| *cls* | cardiolipin synthase | 0 | 1 | 0 |
| *psd* | phosphatidylserine decarboxylase | 1 | 0 | 1 |
| *pemt* | phosphatidylethanolamine methyltransferase | 1 | 1 | 1 |
| **Menaquinone biosynthesis (Uniquinone and other terpenoid-quinone biosynthesis)** |  |  |  |  |
| *UbiA* | 4-hydroxybenzoate polyprenyltransferase | 1 | 1 | 1 |
| *UbiB* | ubiquinone biosynthesis protein | 0 | 1 | 0 |
| *UbiE* | demethylmenaquinone methyltransferase / 2-methoxy-6-polyprenyl-1,4-benzoquinol methylase | 1 | 2 | 1 |
| *UbiG* | 2-polyprenyl-6-hydroxyphenyl methylase / 3-demethylubiquinone-9 3-methyltransferase | 1 | 1 | 1 |
| *UbiH* | 2-octaprenyl-6-methoxyphenol hydroxylase | 0 | 2 | 2 |
| *UbiX* | flavin prenyltransferase | 1 | 1 | 0 |

**Table S5**. Genes of key features related to wastewater treatment among strains CPCC 101601^T^, CPCC 101403^T^, and CPCC 100767.

| **Caterogy** | **Gene** | **Product** | **Biological role** | **Gene copy number of** | | |
| --- | --- | --- | --- | --- | --- | --- |
|  |  |  |  | **CPCC 101601^T^** | **CPCC 101403^T^** | **CPCC 100767** |
| Denitrification | *nar* | nitrate reductase | nitrate reduction | 0 | 5 | 1 |
| Denitrification | *nir* | nitrite reductase | nitrate reduction | 0 | 0 | 1 |
| Denitrification | *nor* | nitric oxide reductase | nitric oxide reduction | 0 | 0 | 2 |
| Denitrification | *nos* | nitrous-oxide reductase | nitrous-oxide reduction | 0 | 0 | 0 |
| Poly-P biosynthesis | *ppk* | polyphosphate kinase | Poly-P biosynthesis | 3 | 4 | 4 |
| PHA biosysthesis | *phaC* | poly[(R)-3-hydroxyalkanoate] polymerase subunit | polymerase | 0 | 1 | 1 |
| PHA biosysthesis | *phaZ* | poly(3-hydroxybutyrate) depolymerase | PHA depolymerase | 1 | 1 | 1 |
| AM metabolism | *gmaS* | glutamate---methylamine ligase | GMA synthetase | 1 | 1 | 1 |
| AM metabolism | *mauA* | methylamine dehydrogenase light chain | MMA dehydrogenase | 0 | 1 | 0 |
| AM metabolism | *mgsA* | methylamine---glutamate N-methyltransferase subunit A | NMG synthase | 1 | 1 | 1 |
| AM metabolism | *mgsB* | methylamine---glutamate N-methyltransferase subunit B | NMG synthase | 1 | 1 | 1 |
| AM metabolism | *mgsC* | methylamine---glutamate N-methyltransferase subunit C | NMG synthase | 1 | 1 | 1 |
| AM metabolism | *mgdA* | methylglutamate dehydrogenase subunit A | NMG dehydrogenase | 2 | 1 | 2 |
| AM metabolism | *mgdB* | methylglutamate dehydrogenase subunit B | NMG dehydrogenase | 2 | 1 | 2 |
| AM metabolism | *mgdC* | methylglutamate dehydrogenase subunit C | NMG dehydrogenase | 2 | 1 | 2 |
| AM metabolism | *mgdD* | methylglutamate dehydrogenase subunit D | NMG dehydrogenase | 1 | 0 | 1 |
| AM metabolism | *mttB* | trimethylamine---corrinoid protein Co-methyltransferase | TMA methyltransferase | 10 | 0 | 3 |
| AM metabolism | *tmm* | trimethylamine monooxygenase | TMA monooxygenase | 1 | 1 | 1 |
| Nitrogen fixation | *nifH* | Nitrogenases | Nitrogen fixation | 1 | 0 | 1 |
| ALA biosysthesis | *hemA* | ALA synthases | 5-ALA synthase | 1 | 1 | 1 |

**Table S6**. Secondary metabolite biosynthesis gene clusters predicted from the strains CPCC 101601^T^, CPCC 101403^T^, and CPCC 100767.

| **Regoin** | **Contig ID** | **Type** | **From** | **To** | **Secondary metabolite synthesis gene cluster** | **Similarity** |
| --- | --- | --- | --- | --- | --- | --- |
| **CPCC 100767** | |  |  |  |  |  |
| Region 1.1 | JAVCWG010000001.1 | NRP-metallophore,NRPS | 115,983 | 165,999 | cupriachelin | 17% |
| Region 1.2 | JAVCWG010000001.1 | T1PKS, NRPS-like | 410,484 | 462,461 |  |  |
| Region 1.3 | JAVCWG010000001.1 | hserlactone | 627,523 | 648,110 |  |  |
| Region 1.4 | JAVCWG010000001.1 | redox-cofactor | 651,979 | 688,615 | JBIR-06 | 16% |
| [Region 12.1](applewebdata://C8FF3669-617B-449A-BE9A-D5A8D7918B8F#RANGE!r12c1) | JAVCWG010000002.1 | [terpene](https://docs.antismash.secondarymetabolites.org/glossary/#terpene) | 254,548 | 275,303 |  |  |
| Region 27.1 | JAVCWG010000006.1 | betalactone | 154,459 | 179,706 |  |  |
| Region 27.1 | JAVCWG010000006.1 | betalactone | 154,459 | 179,706 |  |  |
| Region 28.1 | JAVCWG010000009.1 | RiPP-like | 95,170 | 106,069 |  |  |
| **CPCC 101403^T^** |  |  |  |  |  |  |
| Region 1.1 | JAVRQI010000001.1 | NRPS-like,T1PKS | 144,772 | 196,670 |  |  |
| Region 1.2 | JAVRQI010000001.1 | hserlactone | 211,943 | 232,533 |  |  |
| Region 3.1 | JAVRQI010000011.1 | NRP-metallophore,redox-cofactor | 66,192 | 130,557 | parabactin | 100% |
| Region 4.1 | JAVRQI010000012.1 | thioamitides | 22,579 | 45,148 |  |  |
| Region 12.1 | JAVRQI010000002.1 | hydrogen-cyanide | 252,786 | 266,748 |  |  |
| Region 23.1 | JAVRQI010000003.1 | thioamide-NRP | 246,709 | 287,962 |  |  |
| Region 23.2 | JAVRQI010000003.1 | T3PKS | 288,061 | 313,817 | oryzanaphthopyran A/oryzanaphthopyran B/oryzanaphthopyran C/oryzanthrone A/oryzanthrone B/chlororyzanthrone A/chlororyzanthrone B | 6% |
| Region 24.1 | JAVRQI010000030.1 | hserlactone | 1 | 20,555 |  |  |
| Region 41.1 | JAVRQI010000005.1 | hserlactone | 112,927 | 133,682 |  |  |
| Region 48.1 | JAVRQI010000007.1 | NRPS-like | 177,303 | 219,747 |  |  |
| Region 48.2 | JAVRQI010000007.1 | NRPS-like | 253,729 | 276,690 |  |  |
| **CPCC 101601^T^** |  |  |  |  |  |  |
| Region 5.1 | JAVDBT010000013.1 | hserlactone | 112,203 | 132,790 |  |  |
| Region 23.1 | JAVDBT010000003.1 | terpene | 191,114 | 211,863 |  |  |
| Region 41.1 | JAVDBT010000006.1 | NRPS-like,T1PKS | 30,693 | 82,540 | diutan polysaccharide | 16% |
